# Thigmotaxis in a virtual human open field test

**DOI:** 10.1038/s41598-021-85678-5

**Published:** 2021-03-23

**Authors:** Daniel Gromer, Dominik P. Kiser, Paul Pauli

**Affiliations:** 1 Department of Psychology (Biological Psychology, Clinical Psychology, and Psychotherapy), University of Würzburg, Würzburg, Germany; 2 Center of Mental Health, Medical Faculty, University of Würzburg, Würzburg, Germany

**Keywords:** Anxiety, Human behaviour

## Abstract

Animal models are used to study neurobiological mechanisms in mental disorders. Although there has been significant progress in the understanding of neurobiological underpinnings of threat-related behaviors and anxiety, little progress was made with regard to new or improved treatments for mental disorders. A possible reason for this lack of success is the unknown predictive and cross-species translational validity of animal models used in preclinical studies. Re-translational approaches, therefore, seek to establish cross-species translational validity by identifying behavioral operations shared across species. To this end, we implemented a human open field test in virtual reality and measured behavioral indices derived from animal studies in three experiments ($\textit{N}=31$, $\textit{N}=30$, and $\textit{N}=80$). In addition, we investigated the associations between anxious traits and such behaviors. Results indicated a strong similarity in behavior across species, i.e., participants in our study—like rodents in animal studies—preferred to stay in the outer region of the open field, as indexed by multiple behavioral parameters. However, correlational analyses did not clearly indicate that these behaviors were a function of anxious traits of participants. We conclude that the realized virtual open field test is able to elicit thigmotaxis and thus demonstrates cross-species validity of this aspect of the test. Modulatory effects of anxiety on human open field behavior should be examined further by incorporating possible threats in the virtual scenario and/or by examining participants with higher anxiety levels or anxiety disorder patients.

## Introduction

Anxiety disorders are among the most prevalent mental disorders^1^, have a high burden of disease^2^, and lead to considerable costs in the health care system^3^. Psychotherapy is an effective treatment for anxiety disorders^4,5^; however, a considerable proportion of patients does not reach remission^6^ and some patients experience a relapse even after successful treatment^7^. Likewise, although pharmacotherapy has been found effective in the treatment of anxiety disorders, meta-analyses indicate only small to moderate effect sizes in comparison to placebo^8–10^. One possible explanation for the limited effectiveness of current treatments is that mechanisms underlying anxiety disorders are not well understood. Indeed, although the last decades have brought immense new insights into the neurobiological underpinnings of threat-related behaviors^11^ and (pathological) anxiety^12,13^, this has not yet led to new treatments or an improvement of current treatments for anxiety disorders^14,15^. In the case of psychiatric drug development research, animal models are used in preclinical studies to screen for possible new compounds that affect a desired target, e.g., anxiety. Effects of drugs on targets like anxiety are usually inferred from behaviors observed in specifically designed paradigms. However, candidate compounds from preclinical studies often end up failing in clinical studies in humans (the likelihood of approval for psychiatric drugs entering Phase I trials was 6.2%, which is the second-lowest among all disease areas^16^). Indeed, no new drugs for anxiety disorders have been approved by the EMA or FDA in the 2010s^17^. One possible reason for the lack of success in translating findings from animal models to human pathological anxiety is the unknown predictive and cross-species translational validity of the animal models used in preclinical studies^18,19^.

Animal paradigms widely used in anxiety research are the open field test (OFT), the elevated plus-maze (EPM), and the light/dark-test, among others^20–22^. The OFT was introduced by Hall in 1934^23^ to measure emotional behavior in rodents. In the test, a rodent is placed in a novel open space (e.g., a square 50 $\times$ 50 $\times$ 30 cm box) for a fixed period of time (e.g., 10 min) while its behavior is measured (e.g., center avoidance, total distance covered, defecation, grooming, or rearing)^24^. A central underlying assumption is that rodents avoid open spaces due to a fear of (aerial) predators^25^, rendering thigmotaxis–the movement along walls–an anxiety-like behavior^26^. The findings that anxiolytic drugs like benzodiazepines or $\text {5-HT}_{\text {1A}}$ receptor agonists typically increase the time spent in the (otherwise avoided) center of the open field^27^ support the assumption that behavioral OFT indices (e.g., center avoidance) are valid indicators of anxiety and have predictive validity for detecting anxiolytic effects (e.g.,^26^). However, the OFT is not sensitive to other drugs used in the treatment of anxiety disorders, particularly selective serotonin reuptake inhibitors^27^, which are currently the first-line pharmacotherapy for anxiety disorders (see, e.g., the German treatment guidelines for anxiety disorders^28^).

These conflicting results, together with the problems in discovering new anxiolytic medications in preclinical research^15,16^, challenge the validity of the rodent OFT as an adequate model for pathological anxiety^18,19,27,29^. As validity of animal models is essential for preclinical research, Grillon & Ernst (2016)^19^ suggest the use of re-translational approaches, i.e., conducting model tests in humans and comparing the results with animal studies. Cross-species translational validity of preclinical models may be achieved by “maximizing the similarity of the measures of responses across species” (^19^, p. 343).

First re-translational studies suggest the value of this approach. For example, Walz et al.^30^ realized a human open field test on a soccer field, comparing the movement behavior of agoraphobic patients vs. healthy controls and of participants high vs. low in anxiety sensitivity. In accordance with the hypotheses derived from the animal model, all participants exhibited some thigmotaxis, but agoraphobic patients and participants with high anxiety sensitivity showed more movement along the outer region of the open field (i.e., thigmotaxis), entered the center of the open field less frequently, and had a higher latency for the first center entry in comparison to their respective control group. These results suggest a cross-species similarity between animal and human (agoraphobic) open field behavior. Similarly, Biedermann et al.^31^ re-translated the EPM to a human virtual reality test. In accordance with the hypotheses derived from the animal model, open arms induced more anxiety than closed arms, and these anxiety ratings correlated with behavioral avoidance of the open arms. Again, results suggest a possible cross-species similarity in open arm avoidance, with the caveat that the human EPM results seemed to be affected by trait acrophobia^31^, an effect not in accordance with animal EPM behavior, which is thought to be independent of height^32,33^. Further studies have reported initial successes of such re-translational approaches (e.g.,^34–40^). This line of research is valuable, as human preclinical models demonstrating cross-species translational and predictive validity may successfully bridge the gap between animal preclinical research and clinical studies^41^.

The present study aimed at translating the Walz et al.^30^ human OFT to virtual reality for several reasons. Firstly, virtual reality has high ecological validity and simultaneously allows strong laboratory-like experimental control. Secondly, virtual reality allows repeated testing in the laboratory and therefore facilitates studies of underlying mechanisms based on distinct experimental manipulations. Thirdly, experimental repetitions with constant conditions allow controlled comparisons between groups, e.g., various anxiety disorders, as well as repeated testing within groups, e.g., to evaluate treatment effects. Finally, combined testing with various re-translated models assumed to measure the same construct, e.g., the OFT and EPM, would be possible, which may improve test validity.

With the virtual OFT, we investigated human movement behavior in a virtual open field in analogy to animal studies and evaluated whether anxiety traits modulate such behavior. Our hypotheses were: (1) participants prefer to stay in the outer region of the open field as indicated by behavioral indices derived from animal studies; (2) this preference for the outer region is a function of trait anxiety measures of participants, with a stronger preference for the outer region in participants with higher anxiety; (3) participants report more anxiety and arousal in the inner compared to the outer region. To this end, we conducted three experiments in virtual reality, testing different versions of the human OFT.

## Materials and methods

### Sample

Participants in all three experiments were recruited on a local subject recruitment platform at the University of Würzburg. The inclusion criterion was age between 18–60, the exclusion criteria were pregnancy and epilepsy (in subjects or near relatives). The samples in experiments 1, 2, and 3 consisted of $\textit{N}=31$ participants (age: $\textit{M}=29.1$, $\textit{SD}=9.8$; 21 female), $\textit{N}=30$ participants (age: $\textit{M}=25.5$, $\textit{SD}=6.2$; 25 female), and $\textit{N}=80$ participants (age: $\textit{M}=24.8$, $\textit{SD}=5.4$; 58 female), respectively. No participants were excluded from data analysis. Ethical approval for the experimental procedure (virtual reality setup and subjective reports) was obtained for previous studies from the Ethics Committee of the Medical Faculty of the University of Würzburg. All participants gave their written informed consent before the experiments in accordance with the Declaration of Helsinki. Participants received either 9 EUR or course credit for their participation.

### Measures

#### Questionnaires

*State-Trait Anxiety Inventory* (STAI;^42,43^). The STAI is a self-report questionnaire that assesses anxiety as a state and as a trait. The state anxiety subscale consists of 20 items (e.g., “I am worried”) rated on a four-point Likert scale ranging from 1 (“not at all”) to 4 (“very much so”). The trait anxiety subscale consists of 20 items (e.g., “I worry too much over something that really doesn’t matter”), rated on a four-point Likert scale ranging from 1 (“almost never”) to 4 (“almost always”). Participants are asked to rate the statements according to how they currently feel (state) or how they feel in general (trait). Scores for both subscales have a range of 20–80.

*Agoraphobic Cognitions Questionnaire* (ACQ;^44,45^). The ACQ assesses the self-reported frequency of agoraphobic cognitions on two subscales: *loss of control* (e.g., “I will not be able to control myself”) and *physical concerns* (e.g., “I am going to pass out”). The 14 items are rated on a five-point Likert scale ranging from “thought never occurs” to “thought always occurs” and subsequently means are calculated for each subscale and a total score (1–5).

*Mobility Inventory* (MI;^45,46^). The MI is a self-report measure that assesses avoidance of common situations (e.g., supermarkets, open spaces, trains) due to discomfort or anxiety. Avoidance is rated on a five-point Likert scale ranging from “never avoid” to “always avoid” for either being alone or when accompanied with a trusted person. Scores for the subscales *alone* and *accompanied* are calculated by averaging the respective ratings (1–5).

*Anxiety Sensitivity Index—3* (ASI-3;^47,48^). The ASI-3 assess self-reported anxiety sensitivity, i.e., the tendency to interpret symptoms of fear and anxiety as harmful^49^. The questionnaire comprises the three subscales *physical concerns* (e.g., “When I feel pain in my chest, I worry that I’m going to have a heart attack”), *cognitive concerns* (e.g., “When my mind goes blank, I worry there is something terribly wrong with me”), and *social concerns* (e.g., “When I begin to sweat in a social situation, I fear people will think negatively of me”). The 18 items are rated with respect to how strong these apply to participants on a five-point Likert scale ranging from 0 (“very little”) to 4 (“very”). Sum scores for each scale are calculated from six respective items (0–24) in addition to a total score (0–54).

#### Ratings

Anxiety and arousal within the virtual open field were assessed by verbal reports using the questions “how anxious do you feel in this location on a scale of 0–100?” and “how aroused do you feel in this location on a scale of 0–100”. Prior to the experiment, participants were given anchors for 0 (“not at all”) and 100 (“extreme—as strong as I can imagine”). The rating scales were derived from the Subjective Units of Distress Scale (SUDS,^50^).

#### Behavior

**Figure 1:**
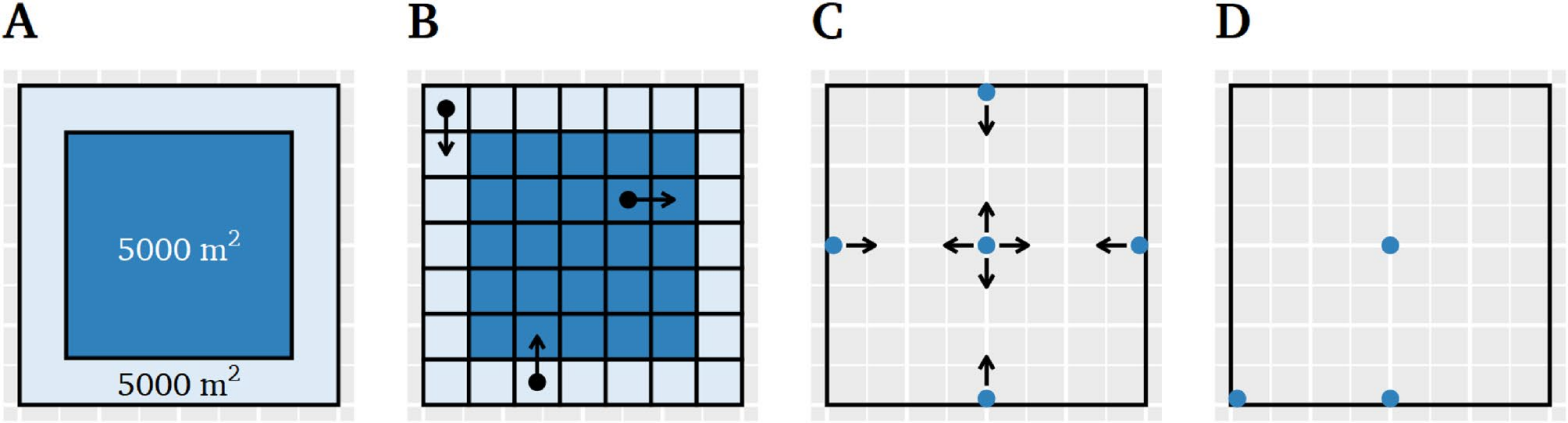
(**A**) Areas of the inner and outer region of the virtual open field. (**B**) Binning used to calculate movement parameters based on line crossings. The arrow in the top left corner illustrates a line crossing counted as thigmotaxis. The arrow in the center region illustrates a line crossing counted as center ambulation. The arrow at the bottom illustrates a line crossing counted as center entry. (**C**) Starting positions. (**D**) Rating positions.

Positioning data of participants in the virtual open field was extracted from the player position in Unreal Engine at a sample rate of 20 Hz and saved to a local text file (see https://github.com/dgromer/ue4-misc/blob/master/LogFileWriter/4.20/LogfileWriter.cpp for the implementation). From the positioning data, we calculated several behavioral indices:

*Time in outer region.* For this index, the open field was subdivided into two areas: an inner and outer region. The area of the inner region was defined to be half of the total area (= 10,000 m^2^ / 2, resulting in a side length of $50\times \sqrt{2}$ m for the inner region). The outer region was defined as the remaining area (i.e., the width of the outer region was $(100 - 50 \times \sqrt{2}) / 2 = 14.64$ m) (see Fig. 1A). The time spent in the outer region (in seconds) was calculated as the sum of positioning data samples within the outer region divided by the sample rate (range 0–900 s). In addition to the time spent in the outer region, the *total path length* in meters (= the sum of Euclidean distances between each two positioning samples) and *mean walking speed* in m/s (= total path length divided by the time spent in the respective region) were calculated for both regions as control variables.

*Proximity to wall.* For this index, the distance to the nearest wall of the open field was calculated for each sample and subsequently averaged across all samples. The proximity to wall index has a range of 0–50 m.

*Thigmotaxis, center entries, and center ambulation.* For these indices, the open field was subdivided into a raster of 7 $\times$ 7 bins (side length of $100 / 7 = 14.29$ m each), resulting in 24 inner bins and 25 outer bins (approximating equal area of inner vs. outer regions) (see Fig. 1B). Thigmotaxis was defined as the number of line crossings within outer region bins, center entries were defined as the number of line crossings from an outer to an inner bin, and center ambulation was defined as the number of line crossings within inner region bins (see also^30^). As we later report the proportion of each line crossing parameter in relation to the total number of line crossings, we further calculated the fourth possible line crossing type: center leaves, defined as a line crossing from an inner to an outer bin.

### Apparatus and software

The experiments ran on a Windows 10 64-bit machine (Intel Core i7-2600k, 16 GB RAM, Samsung Evo 850 SSD, NVIDIA Geforce 970 GTX) and were displayed on a HTC VIVE (HTC, New Taipei City, Taiwan) with a resolution of 1080 $\times$ 1200 pixels per eye. The Lighthouse tracking system of the HTC VIVE was used for positional and rotational tracking of participants. A HTC VIVE Controller was used for movement within the virtual environment. Audio instructions were presented via a HTC VIVE Deluxe Audio Strap.

The experiment and virtual open field were built in Unreal Engine 4.20 (Epic Games, Cary, North Carolina, USA) using the built-in virtual reality template and assets from the “Paragon: Agora and Monolith Environment” pack. Movement within the virtual environment was possible by either teleportation (using the technique from Unreal Engine’s virtual reality template for motion controllers) or actual walking within the space of the laboratory (about 2.5 m $\times$ 3.5 m). The SteamVR Chaperone system warned participants when getting to close to a real wall.

### Virtual human open field test

**Figure 2:**
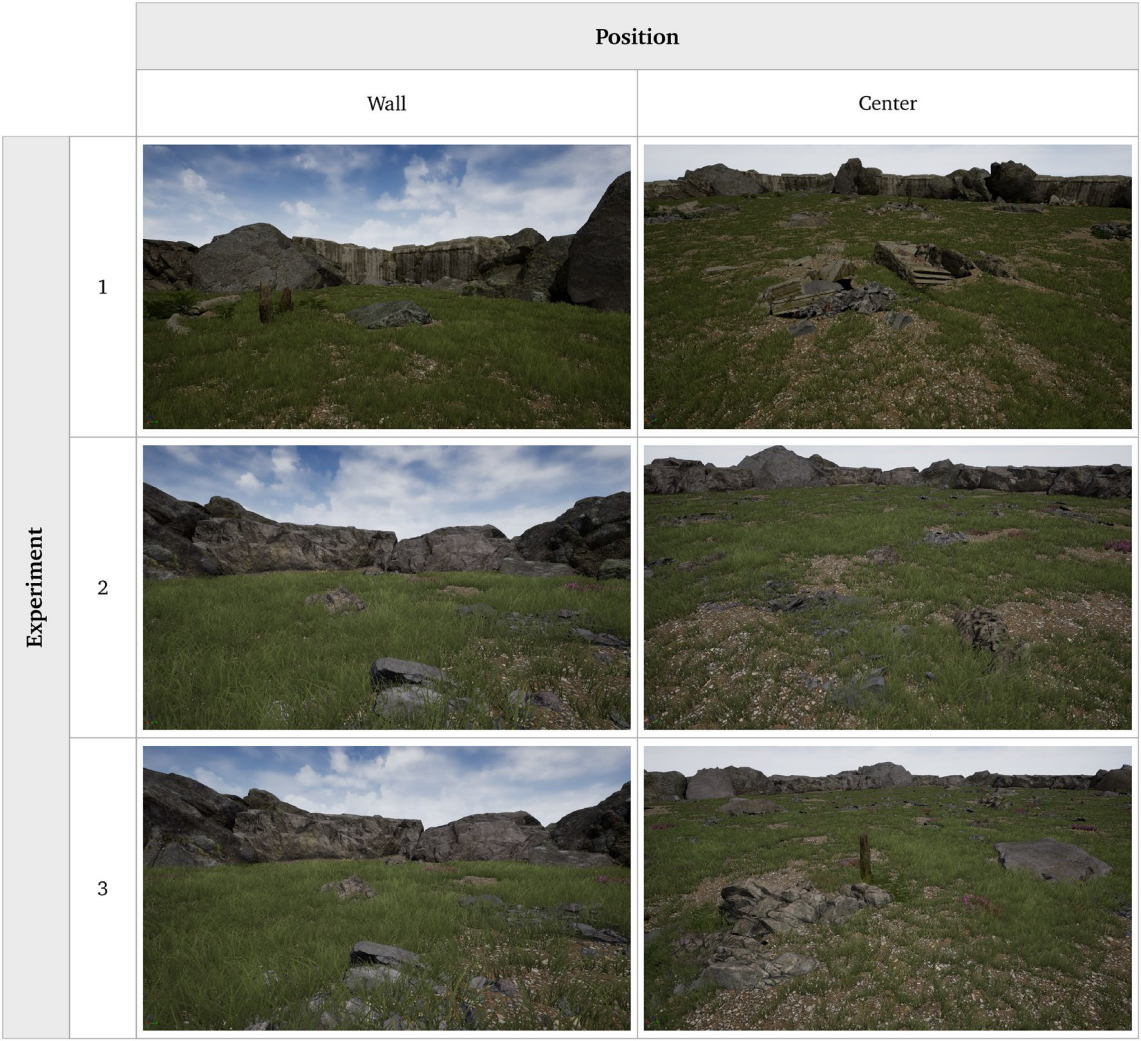
Screenshots depicting the three versions of the virtual human open field test.

For the present study, a virtual open field was built with the goal to translate the original experimental setup to an environment appealing to human subjects. The virtual open field thus consisted of a 100 m $\times$ 100 m grassland with stones and flowers spread across the area. In the three experiments, slightly different versions of the open field were used (see Fig. 2). Modifications were applied successively in an attempt to better control and standardize the effects of open field design on movement behavior. In experiment 1, the outer wall was a mix of concrete-like walls and large rocks, about 3–6 m in height, and parts of ancient concrete structures were spread across the field. In experiment 2 and 3, the outer walls were made of large rocks only, about 2.5–4.5 m in height, and the ancient concrete structures were removed. In experiment 2, only few small rocks were spread across the field. In experiment 3, more and larger rocks and some tree stumps were added across the field.

### Experimental design and procedure

All three experiments were based on a correlational design to investigate associations between anxious traits and movement behavior in the open field.

Upon arrival at the laboratory, participants read an information letter and signed the informed consent. Next, the experimenter explained how participants could use the HTC VIVE Controller to navigate within the virtual environment. Participants were then equipped with the head-mounted display and controller. In the training phase, participants moved through a generic virtual environment collecting blue spheres to get accustomed to the virtual reality and the movement within the virtual environment. After completing the training phase, the display faded to black and participants were teleported to the virtual open field. Participants randomly started in one of eight positions: either in the center looking towards of one of the four walls, or at one of the four walls looking towards the center (see Fig. 1C). When the image faded in, participants received an audio instruction with the task to explore the virtual environment freely. Moreover, participants were asked not to stay in one position for a prolonged time. After 15 min, the display faded to black and three rating trials started. In these trials, participants were successively teleported to three locations (center of the open field, at a wall, and at a corner, see Fig. 1D) in pseudo-randomized order and asked to rate their anxiety and arousal. After the ratings, participants were helped to put off the HMD and filled in questionnaires (demographics, STAI, ACQ, MI, and ASI-3).

### Data analysis

All statistical analyses were conducted with R 3.5.1^51^. The afex package^52^ was used for ANOVA. Confidence intervals for Cohen’s *d* (95%) and partial eta-squared ($\eta ^2_p$) (90%) were calculated with the MBESS package^53,54^. In the ANOVA for anxiety ratings (experiment 3), one participant had to be excluded due to missing data in one of the three ratings.

To test the hypothesis that participants prefer to stay in the outer region of the open field, we used a simulation approach to calculate expected values for the behavioral indices based on random moving agents. These expected values were then used for comparison with the participants’ actual movement behavior. In the following, we will describe the simulation approach; for the acutal implementation, see Supplementary Material 1. As participants used the teleportation technique to move within the virtual environment, such teleportation movements were simulated. The total number of simulated teleportations per run was derived from the actual movement behavior of participants in the three experiments (i.e., the median number of teleportations per participant was 367). Likewise, the teleportation distances were also sampled from the participants’ actual teleportation distances. A simulated run began in one of the eight starting positions of the open field (see Fig. 1C). Then, both a teleportation distance and direction ($\mu = 0^{\circ }$, i.e., straight on, $\sigma = 45^{\circ }$) were sampled and, if the destination was within the area of the open field, the teleportation was executed. Otherwise, a new teleportation destination was sampled. Each simulation ran until reaching the defined number of teleportations. A total of 1,000 simulation runs were conducted and expected values for the behavioral indices were then calculated from this simulated movement data.

**Figure 3:**
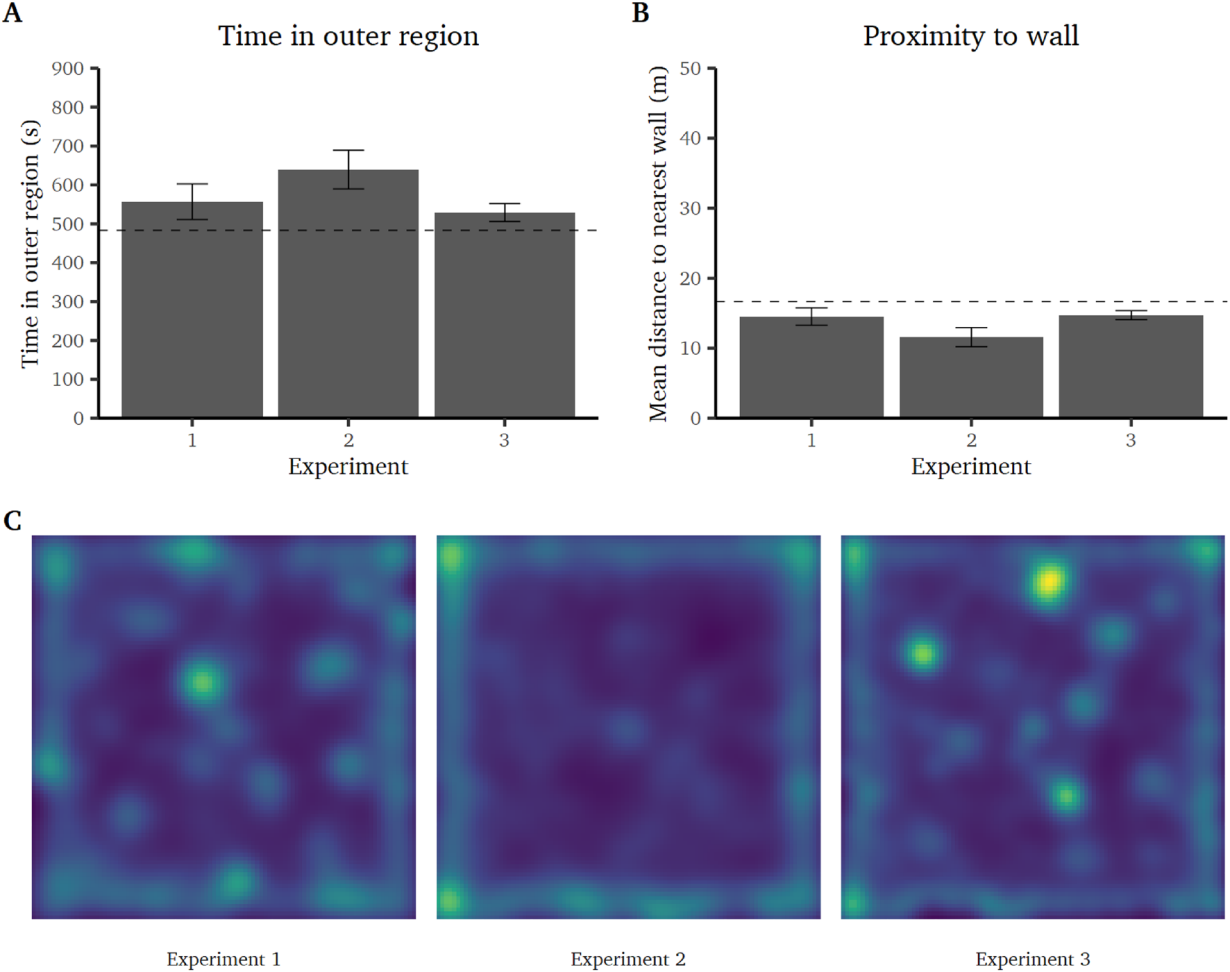
(**A**) Mean time (in seconds) spent in the outer region of the open field in experiments 1–3 (± 95% CI; indicates significance if the CI does not include the expected value of 483.40 s (dashed line)). (**B**) Mean distance (in meters) to the nearest wall of the open field in experiments 1–3 (± 95% CI; indicates significance if the CI does not include the expected value of 16.67 m (dashed line)). (**C**) Heatmaps of the positioning data of all participant in experiments 1–3. Shading indicates the time spent in the respective position across all participants (the brighter, the more time spent in the respective position).

Hypotheses were tested separately by study and not combined, because of systematic differences in movement behavior across the three experiments (see Fig. 3C).

## Results

### Time in the outer region

First, we tested if participants showed a stronger preference for the outer region of the open field than would be expected by random movement. For this purpose, we conducted one-sample t-tests on the time in the outer region (against the expected value $\mu = 483.40$ s). In all three experiments, participants spent more time in the outer region of the open field than expected, experiment 1: $\textit{t}(30)=3.28$, $\textit{p}=.003$, $\textit{d}=0.59$ [0.20; 0.97]; experiment 2: $\textit{t}(29)=6.42$, $\textit{p}<.001$, $\textit{d}=1.17$ [0.70; 1.63]; and experiment 3, $\textit{t}(79)=3.94$, $\textit{p}<.001$, $\textit{d}=0.44$ [0.21; 0.67] (see Fig. 3A). Moreover, in all three experiments, participants spent more time in the outer compared to the inner region (i.e., comparing against $\mu = 450$ s). In addition, we compared the walking distances and walking speeds in the inner vs. outer region of the open field using paired t-tests. The performed walking distances were larger in the outer compared to the inner region of the open field, experiment 1: $\textit{t}(30)=2.18$, $\textit{p}=.037$, $\textit{d}=0.39$ [0.02; 0.75]; experiment 2: $\textit{t}(29)=5.33$, $\textit{p}<.001$, $\textit{d}=0.97$ [0.53; 1.40]; and experiment 3: $\textit{t}(79)=4.83$, $\textit{p}<.001$, $\textit{d}=0.54$ [0.30; 0.77], but the walking speed was higher in the inner compared to the outer region of the open field, experiment 1: $\textit{t}(30)=-3.16$, $\textit{p}=.004$, $\textit{d}=-0.57$ [− 0.94; − 0.18]; experiment 2: $\textit{t}(29)=-4.29$, $\textit{p}<.001$, $\textit{d}=-0.78$ [− 1.19; − 0.37]; and experiment 3: $\textit{t}(79)=-4.85$, $\textit{p}<.001$, $\textit{d}=-0.54$ [− 0.78; − 0.31].

### Proximity to wall

In addition to analyzing the time in the outer region, we tested if participants were, on average, closer to the wall then what would be expected by random movement. One-sample t-tests (against $\mu = 15.61$ m) showed that, in experiments 2 and 3, participants preferred to stay closer to the wall, experiment 2: $\textit{t}(29)=-6.02$, $\textit{p}<.001$, $\textit{d}=-1.10$ [− 1.55; − 0.64]; and experiment 3: $\textit{t}(79)=-2.75$, $\textit{p}=.007$, $\textit{d}=-0.31$ [− 0.53; − 0.08]. In experiment 1, participants did not stay closer to the wall than expected, $\textit{t}(30)=-1.77$, $\textit{p}=.086$, $\textit{d}=-0.32$ [− 0.68; 0.04] (see Fig. 3B).

### Line crossing parameters

**Figure 4:**
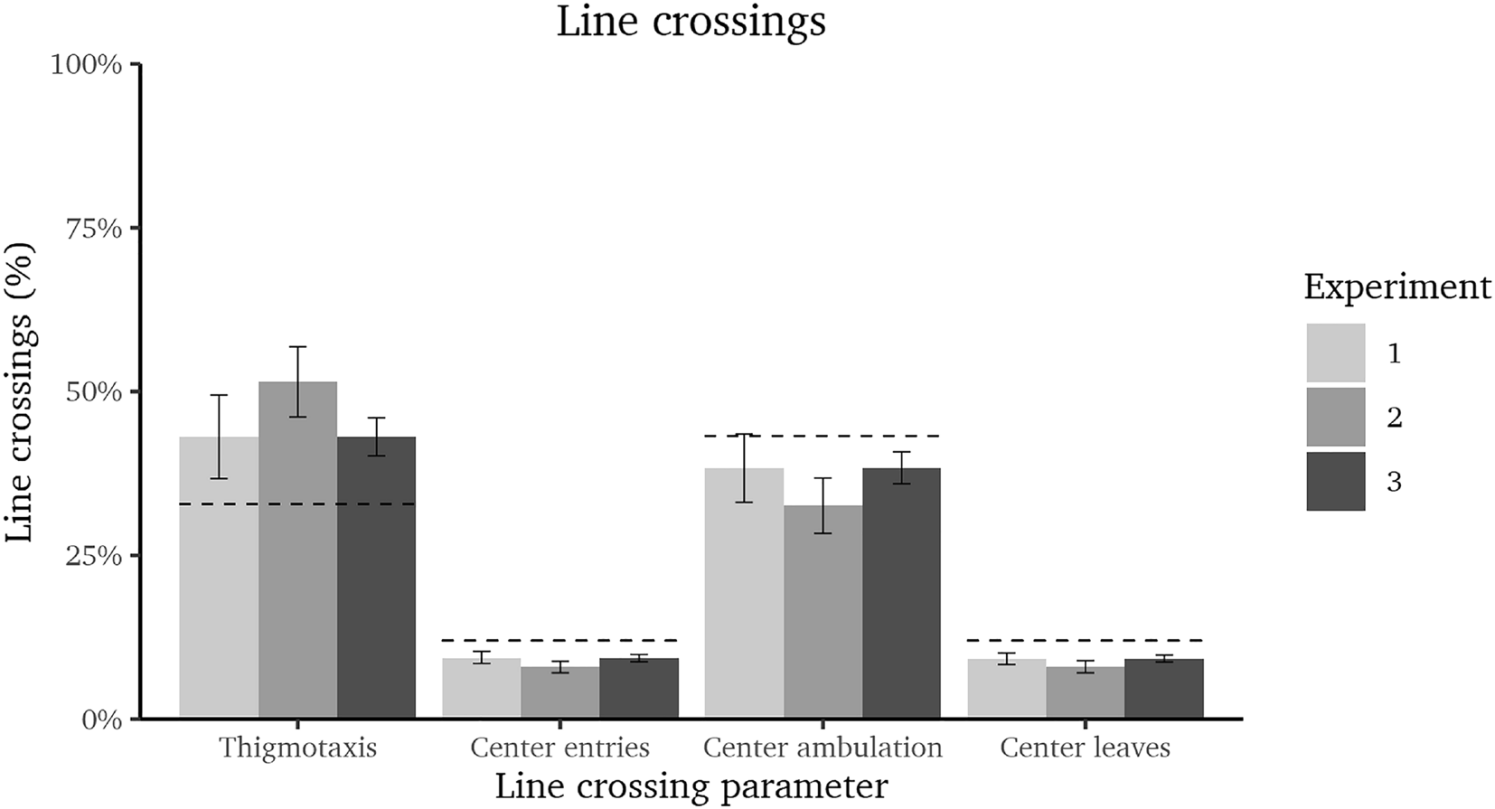
Proportion of thigmotaxis, center entries, center ambulation, and center leaves in total line crossings (± 95% CI; indicates significance if the CI does not include the respective expected value (dashed line)).

To compare the participants’ actual movement behavior (in terms of line crossings, see Fig. 4) with what would have been expected by random movement, we conducted a series of one-sample t-tests.

For thigmotaxis, one-sample t-tests (against $\mu = 32.84$%) showed that, in all three experiments, participants moved more along the wall then would have been expected by chance, experiment 1: $\textit{t}(30)=3.29$, $\textit{p}=.003$, $\textit{d}=0.59$ [0.20; 0.97]; experiment 2: $\textit{t}(29)=7.12$, $\textit{p}<.001$, $\textit{d}=1.30$ [0.80; 1.78]; and experiment 3: $\textit{t}(79)=7.04$, $\textit{p}<.001$, $\textit{d}=0.79$ [0.53; 1.04].

For center entries, one-sample t-tests (against $\mu = 11.97\%$) showed that, in all three experiments, participants showed less center entries than would have been expected by random movement, experiment 1: $\textit{t}(30)=-5.73$, $\textit{p}<.001$, $\textit{d}=-1.03$ [-1.46; $-$0.59]; experiment 2: $\textit{t}(29)=-9.27$, $\textit{p}<.001$, $\textit{d}=-1.69$ [$-$2.25; $-$1.12]; and experiment 3: $\textit{t}(79)=-9.66$, $\textit{p}<.001$, $\textit{d}=-1.08$ [$-$1.35; $-$0.80].

For center ambulation, one-sample t-tests (against $\mu = 43.21\%$) showed that, in experiments 2 and 3, participants moved less across the center of the open field than would have been expected by random movement, experiment 2: $\textit{t}(29)=-5.16$, $\textit{p}<.001$, $\textit{d}=-0.94$ [$-$1.37; $-$0.50]; and experiment 3: $\textit{t}(79)=-3.98$, $\textit{p}<.001$, $\textit{d}=-0.44$ [$-$0.67; $-$0.21]. In experiment 1, participants center ambulation behavior did not significantly differ from the expected values, $\textit{t}(30)=-1.93$, $\textit{p}=.063$, $\textit{d}=-0.35$ [$-$0.71; 0.02].

For center leaves, one-sample t-tests (against $\mu = 11.97\%$) showed that, in all three experiments, participants left the center less frequently than would have been expected by random movement, experiment 1: $\textit{t}(30)=-6.43$, $\textit{p}<.001$, $\textit{d}=-1.15$ [$-$1.61; $-$0.69]; experiment 2: $\textit{t}(29)=-8.92$, $\textit{p}<.001$, $\textit{d}=-1.63$ [$-$2.17; $-$1.07]; and experiment 3: $\textit{t}(79)=-10.18$, $\textit{p}<.001$, $\textit{d}=-1.14$ [$-$1.42; $-$0.85].

### Relationships between anxiety traits and behavior

Correlational analyses were conducted to investigate associations between anxiety traits and movement patterns within the open field. As some of the behavioral parameters are related by design, we conducted the correlational analyses only with time in the outer region, thigmotaxis, and center entries. For each anxiety measure (STAI and the subscales of ASI-3, ACQ, and MI), the correlations with behavioral parameters were calculated and are reported in Tables 1, 2 and 3. Applying an experiment-wise Bonferroni correction ($\alpha =\frac{.05}{24}=.002$), non of the correlations reached significance.

Looking only at the direction of the correlations, there were significantly more correlation coefficients indicating more center avoidance with higher anxiety (46 out of 72), $\chi ^2(1)=5.56$, $\textit{p}=.018$.

In sum, this analysis indicates that there might be an association between anxiety traits and movement behavior with in the open field; however, results were mostly not consistent across the experiments and correlation coefficients were mostly very small.

**Table 1:** Correlations between anxiety measures and time in outer region.

	Experiment 1		Experiment 2		Experiment 3	
	*r*	*p*	*r*	*p*	*r*	*p*
STAI Trait	.23	.213	− .01	.971	.02	.864
ASI-3 Physical Concerns	.15	.433	.07	.706	– .06	.570
ASI-3 Cognitive Concerns	.07	.714	.06	.740	.09	.451
ASI-3 Social Concerns	.21	.258	− .11	.548	.11	.332
ACQ Physical Concerns	.06	.745	.06	.771	.27	.014
ACQ Loss of Control	.13	.500	.02	.908	− .07	.543
Mobility Inventory Accompanied	.17	.348	.05	.792	.06	.592
Mobility Inventory Alone	.06	.758	.07	.721	.14	.207

STAI = State-Trait Aanxiety Inventory; ASI-3 = Anxiety Sensitivity Index—3, ACQ = Acoraphobic Cognitions Questionnaire, MI = Mobility Inventory.

**Table 2:** Correlations between anxiety measures and thigmotaxis.

	Experiment 1		Experiment 2		Experiment 3	
	*r*	*p*	*r*	*p*	*r*	*p*
STAI Trait	− .14	.447	− .11	.554	.28	.011
ASI-3 Physical Concerns	.02	.894	.14	.472	− .01	.895
ASI-3 Cognitive Concerns	− .11	.570	− .11	.545	.24	.033
ASI-3 Social Concerns	.11	.546	− .38	.040	.18	.118
ACQ Physical Concerns	.07	.726	.35	.061	.16	.168
ACQ Loss of Control	− .15	.416	− .24	.199	.04	.720
Mobility Inventory Accompanied	.04	.844	.04	.839	.08	.482
Mobility Inventory Alone	− .12	.538	.01	.966	.29	.010

STAI = State-Trait Anxiety Inventory; ASI-3 = Anxiety Sensitivity Index—3, ACQ = Acoraphobic Cognitions Questionnaire, MI = Mmobility Inventory.

**Table 3:** Correlations between anxiety measures and center entries.

	Experiment 1		Experiment 2		Experiment 3	
	*r*	*p*	*r*	*p*	*r*	*p*
STAI Trait	− .49	.005	.01	.976	.30	.008
ASI-3 Physical Concerns	− .23	.207	.29	.125	− .01	.930
ASI-3 Cognitive Concerns	− .23	.204	.10	.594	.15	.172
ASI-3 Social Concerns	− .09	.626	.02	.903	− .00	.992
ACQ Physical Concerns	− .10	.587	.45	.012	− .07	.555
ACQ Loss of Control	− .25	.180	.06	.752	.09	.437
Mobility Inventory Accompanied	− .07	.699	.10	.606	.04	.732
Mobility Inventory Alone	− .26	.165	.21	.260	.24	.033

STAI = State-Trait Anxiety Inventory; ASI-3 = Anxiety Sensitivity Index—3, ACQ = Acoraphobic Cognitions Questionnaire, MI = Mobility Inventory.

### Ratings

**Figure 5:**
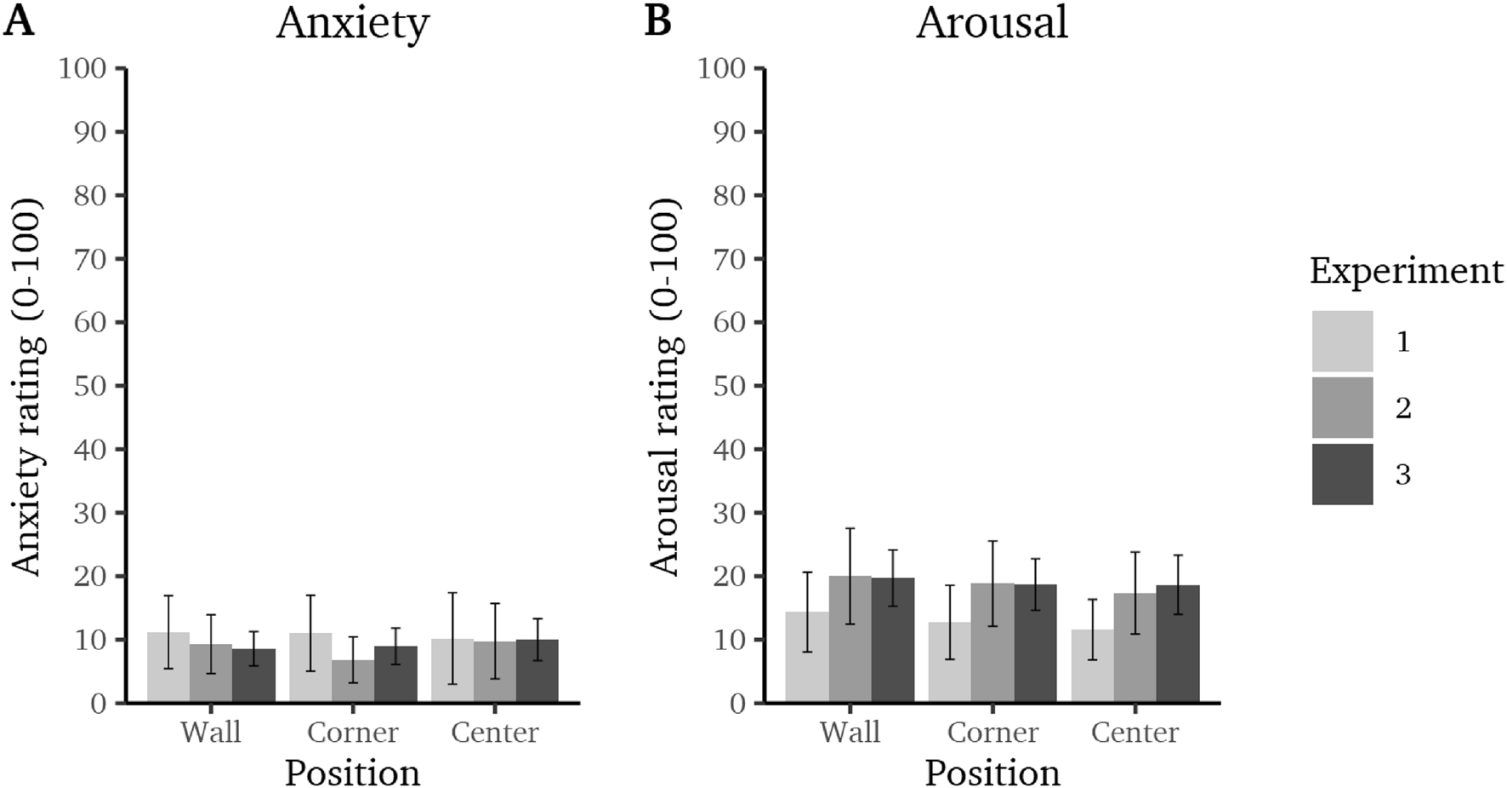
(**A**) Mean ratings of anxiety and (**B**) arousal at the center, corner, and wall of the open field in experiments 1–3 (± 95% CI).

Repeated-measures ANOVA (with Greenhouse-Geisser correction of degrees of freedom if assumption of sphericity was violated) were used to compare the anxiety and arousal ratings at the center, corner, and wall positions respectively for each study. In all three experiments, the anxiety ratings did not differ between positions, experiment 1: $\textit{F}(2,\,60)=0.17$, $\textit{p}=.846$, $\eta ^2_p<.01$ [0.00; 0.04]; experiment 2: $\textit{F}(1.51,\,43.83)=1.13$, $\textit{p}=.317$, $\eta ^2_p=.04$ [0.00; 0.11]; and experiment 3: $\textit{F}(1.75,\,136.84)=0.77$, $\textit{p}=.449$, $\eta ^2_p<.01$ [0.00; 0.04] (see Fig. 5A). Likewise, the arousal ratings did not differ between positions: experiment 1, $\textit{F}(2,\,60)=2.16$, $\textit{p}=.124$, $\eta ^2_p=.07$ [0.00; 0.15]; experiment 2: $\textit{F}(1.53,\,44.47)=0.55$, $\textit{p}=.533$, $\eta ^2_p=.02$ [0.00; 0.08]; and experiment 3: $\textit{F}(2,\,158)=0.32$, $\textit{p}=.728$, $\eta ^2_p<.01$ [0.00; 0.02] (see Fig. 5B).

## Discussion

The present study explored human movement behavior in a virtual OFT and investigated three research questions: firstly, whether humans—analogous to rodents—show a preference for the outer region of the open field; secondly, whether this preference is moderated by anxious traits; and thirdly, whether humans report more anxiety and arousal in the inner compared to the outer region of the open field.

### Preference for the outer region

Regarding the hypothesis that humans–like rodents–spend more time in the outer compared to the center region of the open field, results from all behavioral indices (time in the outer region, average proximity to the nearest wall, thigmotaxis, center entries, and center ambulation) showed a clear preference for the outer region (with only proximity to the nearest wall and center ambulation not reaching significance in one of the three experiments). These findings were consistent across all three experiments and conform with results from rodent studies (e.g.,^55–58^) and a real human open field study^30^.

Quantitative comparisons regarding the time spent in the outer or center regions between rodent studies and the current study are, however, not trivial, as the OFT is a non-standardized test^24,59^ with differing center region areas (e.g., 28% of total area^57^, vs. 11.1% of total area^58^). A descriptive analysis (see Supplementary Material 2) indicated that participants in our virtual OFT tended to spend a little more time (about 7.1–9.6%) in the center region compared to rodents in animal studies^56–58^; however, one study^55^ examined rodents spending about 0.5% more time in the center region than participants in the current study. Moreover, participants in the current virtual OFT descriptively spent less time (about 15.1%) in the center region than participants in the real-life OFT^30^.

In sum, we conclude that thigmotaxis is a cross-species behavior, i.e., is shared among rodents and humans, which can be assessed reliably in humans with a virtual OFT paradigm. However, the quantitative comparisons suggest that the virtual OFT elicits somewhat more thigmotaxis in humans than a real OFT.

### Associations between anxiety and behavior

Correlational analyses between center avoidance and trait measures of anxiety revealed only limited support for the hypothesis that anxiety modulates thigmotaxis and related behaviors in a virtual human OFT. Correlation coefficients were mostly small (and thus statistically not significant) and not consistent across the three experiments. However, we also noted that overall, most correlation coefficients indicated more center avoidance with higher anxiety. This observation suggests a possible modulatory role of anxiety on human behavior in a virtual OFT, which, however, has to be demonstrated conclusively by improved study designs. In fact, the human real-life OFT by Walz et al. (2016)^30^ showed large effect sizes for the comparison of agoraphobic patients vs. healthy controls and of participants high vs. low in anxiety sensitivity with regards to center avoidance measures. Several possible explanations for the weak effects in the present experiments have to be discussed.

First, the samples in the present study were not stratified for the whole range of the trait anxiety measures. This *restriction of range* problem can cause an underestimation of true correlation^60^ and is a possible reason why the present study might have failed to observe a true population effect. In addition, strong increases in thigmotaxis (or center avoidance in general) as a defensive behavior might only occur at a certain degree of anxiety. If thigmotaxis is an evolutionary-conserved adaptive response to open spaces shared across species^30^, it should be mediated by phylogenetically ancient circuits in the brain and increases in anxiety could lead to an activation of such circuits^61,62^. In the real-life OFT, where effects of anxiety on behavior could be found, Walz et al. (2016)^30^ examined agoraphobic patients and persons high in anxiety sensitivity, the latter with an average ASI-3 total score of 31.33 (*SD* = 4.54) compared to average ASI-3 total scores of 18.00 (*SD* = 12.94), 18.90 (*SD* = 12.15), and 17.48 (*SD* = 10.89) in the present experiments. However, by using an extreme group design, the large effect sizes reported by Walz et al. (2016) might have also overestimated the true effect^63^. Animal studies support the view that anxiety has only a moderate or weak effect on open field behavior as the effect of anxiolytic drugs on rodent thigmotactic behavior is limited. For example, only 56% of studies using benzodiazepines detected an effect of the drug on reducing thigmotactic behavior (^27^; and a more recent study^57^). If the population effect of anxiety on open field behavior is indeed moderate or small, the human OFT might only differentiate between high and low levels of anxiety, but not between smaller increments. Future studies should address this empirically by stratification across the whole range of trait anxiety parameters.

Second, trait anxiety likely modulates behavior via state anxiety in a given situation, i.e., individuals with high levels of trait anxiety are more likely to experience state anxiety in a potentially threatening situation, and this enhanced state anxiety motivates avoidance behavior. However, in the present study, participants reported only little anxiety or arousal even in the inner part of the virtual open field, indicating that they did not experience the situation as threatening. Thus, trait anxiety had little effects on state anxiety, and in consequence, we found only few and small associations between trait anxiety and behavior. Following the *Experimental Psychopathology* approach by Grillon et al. (2019)^41^, anxiety-induction procedures are especially valuable to investigate effects of anxiety on behavioral and cognitive operations. An example for such an anxiety-induction procedure is the threat of shock paradigm, which induces sustained anxiety by informing participants that they will receive unpleasant electrical stimuli during the experiment (e.g.,^64–68^). For example, Kirlic, et al. (2017)^69^ demonstrated that participants with high risk for affective disorder display less exploratory behavior in response to a threat of shock. Using this paradigm in the OFT would offer the possibility to investigate the effects of state anxiety on thigmotaxis as a defensive behavior. Future human OFT studies should experimentally manipulate state anxiety in order to investigate how it mediates the effects of trait anxiety on avoidance behavior. Moreover, it might be necessary to consider design changes to the human OFT to better account for human preferences and aversions. In rodents, the OFT possibly induces a state of anticipatory anxiety (pre-encounter threat)^70^ due to a fear of aerial predators, while the current virtual OFT for humans likely had a very low threat imminence. In rodents, brighter lighting is used to increase the aversiveness of the situation. As is commonly known, rodents are nocturnal animals, while humans are diurnal. It stands thus to reason, that the aversive effect of bright lights in rodents in the open field might rather be reproduced using darkness in humans.

Third, discrepancies between the virtual and the real-life OFT^30^ have to be considered, specifically, differences in locomotion and the field of view. In the present study, the participants had to use teleportation for locomotion within the virtual environment^71^. This method was implemented based on our experience and empirical evidence that teleportation–compared with continuous locomotion–induces relatively little simulator sickness (^72–77^, but see^78,79^). In fact, not a single participant in the current study dropped out of the experiment due to simulator sickness. However, research has shown that locomotion methods differentially affect how users navigate within virtual environments (e.g.,^77,80,81^). It can therefore not be ruled out that the realized “unnatural” locomotion method detrimentally affected open field behavior. In addition, the head-mounted display used in the present study had a field of view of $110^{\circ }$, which does not span the whole human field of view of up to $200^{\circ }$^82^ (as cited in^83^). This restriction of peripheral vision might have influenced the perception of the expanse of the open field and thereby the behavioral response. Technological developments might overcome these shortcomings. For example, CAVE systems (e.g.,^84–86^) can present virtual environments with a wider field of view; and more natural locomotion methods like walking in place on an omnidirectional treadmill or redirected walking might better represent real walking^71^.

Fourth, although center avoidance in the open field is commonly considered an anxiety-like behavior (e.g.,^55–58,87,88^), this simplified interpretation has been criticized as being insufficiently validated^18,59^. Indeed, thigmotaxis has also been described as a spatial orientation strategy used to explore unfamiliar environments^38^. Assuming multiple underlying factors driving the use of thigmotaxis, the influence of anxiety on such behavior might be limited to certain situations, populations, or species. For example, the use of thigmotaxis in rodents as a strategy to avoid predators has strong face validity as staying in an open field increases the likelihood of attacks^89^. Likewise, avoidance of an open field center in agoraphobic patients has strong face validity as this behavior is in accordance with the DSM-5 criteria A.2 (“marked fear or anxiety about [...] [b]eing in open spaces” ) and B (“The situations are avoided [...]”)^90^. However, additional motivations might affect open field behavior too (e.g., foraging or mating in animals; curiosity or sensation seeking in humans), and in consequence, the effects of anxiety on open field behavior may be reduced or covered. Such divergent motivational effects covering anxiety effects might be especially prominent in healthy humans with average levels of anxiety, as examined here.

In fact, we found an influence of open field design on movement behavior (see Supplementary Material 3), which suggests that the amount of objects spread across the open field (e.g., stones, flowers) affects behavior. A possible reason for humans to spend less time in the outer region of an enriched environment may be an increased exploratory motivation or a motivation to reduce boredom. Importantly, such factors may not only be relevant for the comparison of different open field designs but likely also play an important role as inter-individual differences affecting open field behavior. Similarly, implementations of the OFT in animal studies (e.g., regarding size, shape, material, lighting, temperature, definition of zones, or definitions of behavioral parameters) vary considerably (e.g.,^56,91–93^, also^24,59^), and this might be a reason for divergent findings. We conclude that it is probably an oversimplified assumption to equate certain open field behaviors directly with anxiety (see also^18^). Both diverse motivations and diverse open field designs likely affect open field behavior. These factors may play a stronger role in humans than in animals; and within humans, a stronger role in healthy than in high anxious individuals or anxiety patients. Future human OFT studies should try to disentangle and assess the motivations at work besides anxiety by measuring additional state (e.g., boredom, engagement) and trait variables (e.g., sensation seeking).

Fifth, self-reported anxious traits and behaviors may only be loosely coupled, although related to the same underlying latent variable. In this regard, it would be necessary to identify and investigate intermediate processes, for example, associations between defensives behaviors and neural activation patterns with e.g., stylized game-like open field paradigms^94–96^. For future research, it is, however, an important goal to bridge the gap between levels of analysis of latent variables such as anxiety. We believe that virtual reality paradigms play an important role in this endeavor, as they provide the opportunity to investigate complex behaviors with high ecological validity. Until now, diagnoses of mental disorders rely on questionnaires and self-reported symptoms only. To improve diagnostics, it might be an important next step to open up the avenue for psychiatric diagnostics to consider evidence from standardized behavioral tests.

### Conclusion

The present study established a human OFT in virtual reality and demonstrated a cross-species similarity in movement behavior, i.e., participants in our virtual open field exhibited thigmotaxis and showed a preference for the outer region comparable to the behavior of rodents in animal studies. However, evidence for correlations between open field behavior and anxious traits was rather weak and not unequivocal. Thus, the re-translation of the animal OFT to a human virtual OFT was successful by revealing thigmotaxis in humans. Future studies should use the approach to further elaborate what exactly the open field test measures and under which conditions anxiety modulates human open field behavior.

## Supplementary information

Supplementary material 1 (pdf 191 KB)

## Data Availability

The data and analysis scripts can be found at https://osf.io/7untj/.
