# Supplementary material for: Thigmotaxis in a virtual human open field test
Source: Sci Rep. 2021 Mar 23;11:6670. doi: 10.1038/s41598-021-85678-5 (PMC7988123; doi:10.1038/s41598-021-85678-5)
Supplement: Supplementary file 1 — Supplementary material 1 (pdf 191 KB) [file 41598_2021_85678_MOESM1_ESM.pdf]

Supplementary Material 1

This is supplementary material for

> Gromer, D., Kiser, D. P. & Pauli, P. (in press). Thigmotaxis in a virtual human open field test.

The following R code performs a simulation of random walks within the open field (see also the Materials and Methods section in the paper).

```
# install.packages(c("tidyverse", "sf"))
library(tidyverse)
library(sf)

# Download positioning data of participants from the OSF repository
load(url("https://osf.io/bw5kg/download"))
```

```
# This function adds a "dist" column to the data frame containing the
# positioning data of a participant. This column holds the euclidean distance
# between successive positions
calc_dist <- function(data)
{
  # First, we create a column that holds the position (x and y)
  data$pos <- map2(data$x, data$y, ~ c(.x, .y))

  # Function for calculating the euclidean distance between two points a and b
  # where a and b are vectors of length 2 holding x and y values
  `%--%` <- function(a, b)
  {
    dist <- sqrt((b[1] - a[1]) ^ 2 + (b[2] - a[2]) ^ 2)

    if (length(dist) == 0) 0 else dist
  }


  # Second, we calculate the distance between each two samples
  data$dist <- map2_dbl(data$pos, lag(data$pos), ~ .x %--% .y)
  # and remove the position column
  data$pos <- NULL

  data
}

# Add a column containing the euclidean distances between each two positions for
# each participant
logfiles_oft <- map(logfiles_oft, calc_dist)

# Calculate the median number of jumps actually performed across all
# participants (will be used as the number of jumps in the simulation)
n_jumps <-
  logfiles_oft %>%
```

```r
  # Only treat distances > 10 cm as jumps (lower values likely represent head
  # movements etc.) and remove jumps that should not be possible
  map(filter, dist > .1 & dist < 20) %>%
  # Calculate the number of jumps per participant
  map(nrow) %>%
  unlist() %>%
  median()
```

```
## [1] 367
```

```r
# Extract a vector with ALL jump lengths actually performed by participants
# (will be used to sample jump lengths in the simulation)
jump_lengths <-
  logfiles_oft %>%
  bind_rows() %>%
  filter(dist > .1 & dist < 20) %>%
  `$`(dist)
```

```r
# Set a seed for reproducibility
set.seed(20201201)

# Initialize an empty list to hold the simulation results
pos_vec <- vector("list", 1000)
```

```r
# Run 1000 simulations
for (j in seq_along(pos_vec))
{
  # Set the start position for the current simulation run. This is either in the
  # center (0, 0) facing one of the outer four walls, or at one of the outer
  # walls facing the center. Starting positions are equally distributed across
  # the simulation runs.
  if (j <= length(pos_vec) / 2)
  {
    current_pos <- c(0, 0)

    if (j <= length(pos_vec) / 8)
    {
      last_angle <- 0
    }
    else if (j <= length(pos_vec) / 8 * 2)
    {
      last_angle <- 90
    }
    else if (j <= length(pos_vec) / 8 * 3)
    {
      last_angle <- 180
    }
    else
    {
      last_angle <- 270
    }
  }
  else if (j <= length(pos_vec) / 8 * 5)
  {
```

```
  current_pos <- c(-49.5, 0)
  last_angle <- 270
}
else if (j <= length(pos_vec) / 8 * 6)
{
  current_pos <- c(49.5, 0)
  last_angle <- 90
}
else if (j <= length(pos_vec) / 8 * 7)
{
  current_pos <- c(0, -49.5)
  last_angle <- 0
}
else
{
  current_pos <- c(0, 49.5)
  last_angle <- 180
}

# Initialize an empty list to hold the positioning data of the current
# simulation run
pos <- vector("list", n_jumps)

# Simulate jumps
for (i in 1:n_jumps)
{
  # Variable used to determine if the next position has been found
  pos_found <- FALSE

  while (!pos_found) {

    # Sample a jumping distance
    new_pos <- c(0, sample(jump_lengths, 1))

    # Sample a jumping direction with mu = 0 (i.e., keeping straight on) and
    # sd = 45 degrees
    new_angle <- rnorm(1, sd = 45)
    if (abs(new_angle) > 180) new_angle <- 180
    angle <- last_angle + new_angle

    s <- sin(angle * pi / 180)
    c <- cos(angle * pi / 180)

    # Calculate the new position (i.e., rotate the `new_pos` counterclockwise)
    new_pos <- c(new_pos[1] * c - new_pos[2] * s,
                 new_pos[1] * s + new_pos[2] * c)

    # Check if the new position is inside the boundaries of the open field. If
    # not, a new position/angle is sampled.
    if (!any(abs(current_pos + new_pos) > 50)) pos_found <- TRUE

  }
```

```r
    # Apply jump and save in `pos` list
    current_pos <- current_pos + new_pos
    pos[[i]] <- current_pos

    last_angle <- angle
  }

  # Inform which simulation run we just ran
  message(sprintf("Iteration %i", j))

  # Save current simulation run
  pos_vec[[j]] <- pos
}

# Convert simulation results into list of tibbles
sim_res <- pos_vec %>%
  map(transpose) %>%
  map_depth(2, unlist) %>%
  map(set_names, c("x", "y")) %>%
  map(as_tibble)
```

Supplementary Material 2

This is supplementary material for

> Gromer, D., Kiser, D. P. & Pauli, P. (in press). Thigmotaxis in a virtual human open field test.

In this descriptive analysis, we compare the time animals spent in the center region in rodent open field tests with the human data from our current study.

For this purpose, we selected several papers and extracted the relative time (in % of total time) animals in control conditions spent in the center region of the respective open field. Next, for each study, we extracted the area of the center region (in % of the total area) and calculated, for our data, the relative time (in % of total time) participants in our study spent in the respective center region.

In addition to the animal studies, we also compare our data with the Walz et al. (2016) data (see last page).

Comparison with Thompson et al. (2015)

> Thompson, T., Grabowski-Boase, L., & Tarantino, L. M. (2015). Prototypical anxiolytics do not reduce anxiety-like behavior in the open field in C57BL/6J mice. *Pharmacology Biochemistry and Behavior*, *133*, 7–17. doi: 10.1016/j.pbb.2015.03.011

Thompson et al. (2015) used a 43.2 × 43.2 cm open field. The center region had an area of 522.6 cm^2 (= 28% of the total area, see Figure 1 A). Control groups spent 8%, 17%, 12%, and 14% of the total time in the center region, respectively (i.e., 8–17%). Translated to our study, 28% center region corresponds to 52.9 × 52.9 m (see Figure 1 B). Calculating the time spent in this region for our experiments returns: experiment 1: 21.74%; experiment 2: 16.78%; and experiment 3: 22.54% of the total time respectively (see Figure 1 C). The mean difference between Thompson et al. (2015) and the current study was -7.6%.

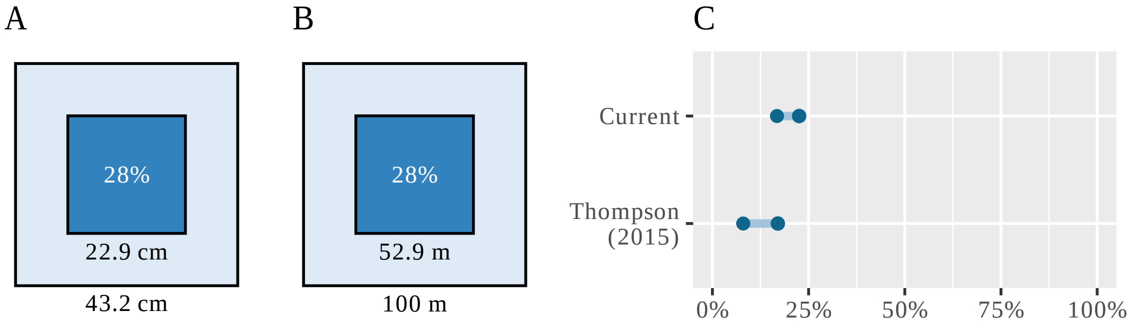

Figure 1: (A) Schematic representation of the Thompson et al. (2015) open field with outer region (light blue) and center region (dark blue). (B) Our open field with the same (relative) size of the center region. (C) Visual comparison of the relative time spent in the center region in Thompson et al. (2015) and our study.

Comparison with Ahrens et al. (2018)

> Ahrens, S., Wu, M. V., Furlan, A., Hwang, G.-R., Paik, R., Li, H., ... Li, B. (2018). A Central Extended Amygdala Circuit That Modulates Anxiety. *The Journal of Neuroscience*, *38*(24), 5567–5583. doi: 10.1523/jneurosci.0705-18.2018

Ahrens et al. (2018) used a 42.5 × 42.5 cm open field. The center region was 21 × 21 cm (= 24% of the total area, see Figure 2 A). The control group spent 80 s out of 900 s in the center region (i.e., 8%). Translated to our study, 24% center region corresponds to 49 × 49 m (see Figure 2 B). Calculating the time spent in this region for our experiments returns: experiment 1: 18.41%; experiment 2: 14.79%; and experiment 3: 19.69% of the total time respectively. The mean difference between Ahrens et al. (2018) and the current study was -9.63%.

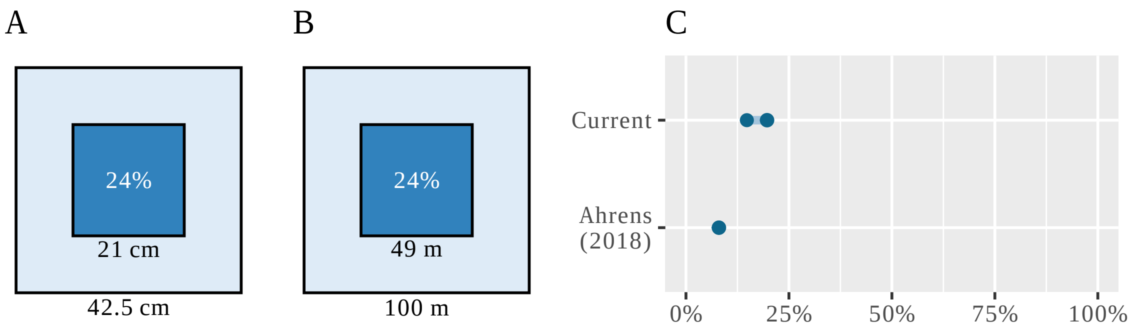

Figure 2: (A) Schematic representation of the Ahrens et al. (2018) open field with outer region (light blue) and center region (dark blue). (B) Our open field with the same (relative) size of the center region. (C) Visual comparison of the relative time spent in the center region in Ahrens et al. (2018) and our study.

Comparison with He et al. (2020)

> He, T., Guo, C., Wang, C., Hu, C., & Chen, H. (2020). Effect of early life stress on anxiety and depressive behaviors in adolescent mice. *Brain and Behavior*, *10*(3), e01526. doi:10.1002/brb3.1526

He et al. (2020) used a 40 × 40 cm open field. The center region was 20 × 20 cm (= 25% of the total area, see Figure 3 A). The control groups spent 65 s out of 300 s (= 21.67%), 50 s out of 300 s (= 16.67%), and 55 s out of 300 s (= 18.33%) in the center region respectively (i.e., 17-22%). Translated to our study, 25% center region corresponds to 50 × 50 m (see Figure 3 B). Calculating the time spent in this region for our experiments returns: experiment 1: 19.40%; experiment 2: 15.32%; and experiment 3: 20.45% of the total time respectively. The mean difference between He et al. (2020) and the current study was 0.5%.

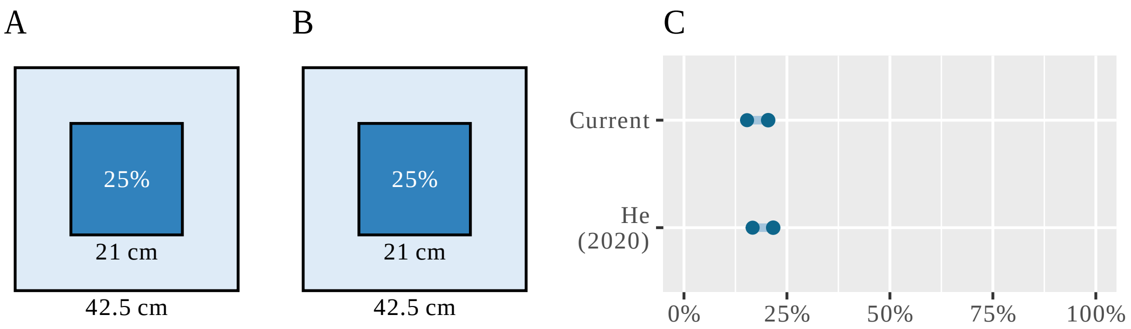

Figure 3: (A) Schematic representation of the He et al. (2020) open field with outer region (light blue) and center region (dark blue). (B) Our open field with the same (relative) size of the center region. (C) Visual comparison of the relative time spent in the center region in He et al. (2020) and our study.

Comparison with Mugdal et al. (2020)

Mudgal, J., Basu Mallik, S., Nampoothiri, M., Kinra, M., Hall, S., Grant, G. D., ... Arora, D. (2020). Effect of coffee constituents, caffeine and caffeic acid on anxiety and lipopolysaccharide-induced sickness behavior in mice. *Journal of Functional Foods*, *64*, 103638. doi: 10.1016/j.jff.2019.103638

Mugdal et al. (2020) used a 30 × 30 cm open field. The center region was 20 × 20 cm (= 11.1% of the total area, see Figure 4 A). The control group spent 6 s out of 300 s (= 2%) in the center region. Translated to our study, 11.1% center region corresponds to 33.3 × 33.3 m (see Figure 4 B). Calculating the time spent in this region for our experiments returns: experiment 1: 10.95%; experiment 2: 6.81%; and experiment 3: 9.53% of the total time respectively. The mean difference between Mugdal et al. (2020) and the current study was -7.1%.

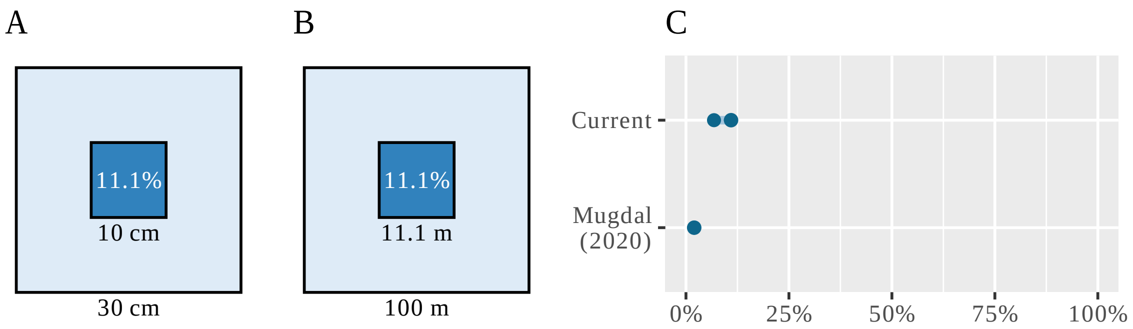

Figure 4: (A) Schematic representation of the Mugdal et al. (2020) open field with outer region (light blue) and center region (dark blue). (B) Our open field with the same (relative) size of the center region. (C) Visual comparison of the relative time spent in the center region in Mugdal et al. (2020) and our study.

Comparison with Walz et al. (2016)

Walz, N., Mühlberger, A., & Pauli, P. (2016). A Human Open Field Test Reveals Thigmotaxis Related to Agoraphobic Fear. *Biological Psychiatry*, *80*(5), 390–397. doi:10.1016/j.biopsych.2015.12.016W

Walz et al. (2016) used a 146 × 79 m open field. The center region was defined as the inner 3 cells of a 5 × 3 grid (= 20% of the total area, see Figure 5 A). The control groups spent 31.33% and 29.13% of the total time in the center region respectively (i.e., 29–31%). Translated to our study, 20% center region corresponds to 44.7 × 44.7 m (see Figure 5 B). Calculating the time spent in this region for our experiments returns: experiment 1: 16.17%; experiment 2: 12.29%; and experiment 3: 16.84% of the total time respectively. The mean difference between Walz et al. (2016) and the current study was 15.13%.

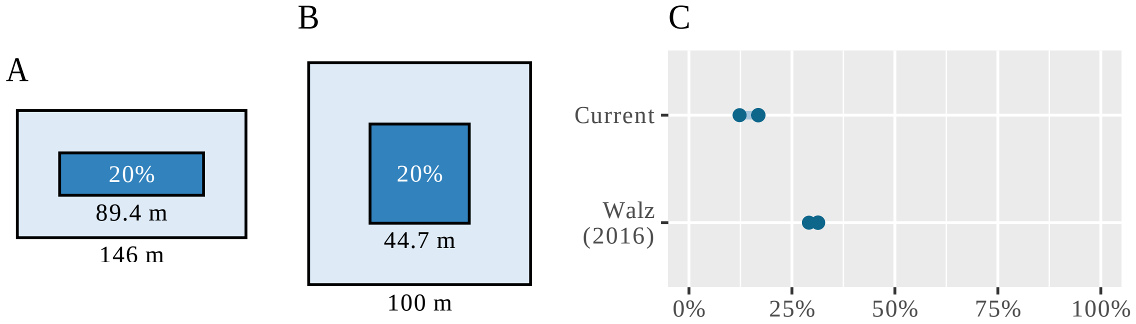

Figure 5: (A) Schematic representation of the Walz et al. (2016) open field with outer region (light blue) and center region (dark blue). (B) Our open field with the same (relative) size of the center region. (C) Visual comparison of the relative time spent in the center region in Walz et al. (2016) and our study.

Supplementary Material 3

This is supplementary material for

Gromer, D., Kiser, D. P. & Pauli, P. (in press). Thigmotaxis in a virtual human open field test.

In this supplementary material, we compare the subjective ratings and movement behavior of participants between experiments 1–3. In the three experiments, the virtual open field was gradually revised and thus slightly modified between experiments. For this reason, we analyze the effects of open field characteristics on ratings and behavior.

Ratings

To test for an effect of experiment on ratings, we conducted separate ANOVAs (with Greenhouse-Geisser correction if assumption of sphericity was violated) on anxiety and arousal ratings with experiment as between factor and position as within factor.

For the anxiety ratings, there was no main effect of experiment, $F(2, 137) = 0.26$, $p = .771$, $\eta_p^2 < .01$ [0.00; 0.03], no main effect of position, $F(1.72, 235.66) = 0.69$, $p = .481$, $\eta_p^2 < .01$ [0.00; 0.02], and no experiment × position interaction, $F(3.44, 235.66) = 0.67$, $p = .592$, $\eta_p^2 < .01$ [0.00; 0.03].

Likewise, for the arousal ratings, there was no main effect of experiment, $F(2, 138) = 1.42$, $p = .246$, $\eta_p^2 =$ .02 [0.00; 0.04], no main effect of position, $F(1.82, 250.57) = 1.75$, $p = .180$, $\eta_p^2 = .01$ [0.00; 0.06], and no experiment × position interaction, $F(3.63, 250.57) = 0.18$, $p = .939$, $\eta_p^2 < .01$ [0.00; 0.00].

Both the anxiety and arousal ratings did not differ between experiments 1–3.

Time in outer region

To test for an effect of experiment on the time spent in the outer region, we conducted an ANOVA with experiment as between factor. There was a significant main effect of experiment, $F(2, 138) = 10.04$, $p < .001$, $\eta_p^2 = .13$ [0.05; 0.21].

Following up on the significant main effect with *post hoc* contrasts (using Tukey's method for correction for multiple comparisons) revealed significant differences between experiment 1 vs. experiment 2, $t(138) = -2.80$, $p = .016$, and between experiment 2 vs. experiment 3, $t(138) = 4.48$, $p < .001$, but not between experiment 1 vs. experiment 3, $t(138) = 1.14$, $p = .489$.

Participants in experiment 2 spent significantly more time in the outer region than participants in both experiments 1 and 3.

Proximity to wall

To test for an effect of experiment on the average distance from the nearest wall, we conducted an ANOVA with experiment as between factor. There was a significant main effect of experiment, $F(2, 138) = 11.24$, $p < .001$, η_p^2 = .14 [0.06; 0.22].

Following up on the significant main effect with *post hoc* contrasts (using Tukey's method for correction for multiple comparisons) revealed significant differences between experiment 1 vs. experiment 2, $t(138) = 3.63$, $p = .001$, and between experiment 2 vs. experiment 3, $t(138) = -4.63$, $p < .001$, but not between experiment 1 vs. experiment 3, $t(138) = -0.29$, $p = .955$.

Participants in experiment 2 were, on average, significantly closer to the wall than participants in both experiments 1 and 3.

Line crossing parameters

To test for an effect of experiment on line crossing parameters, we conducted separate ANOVAs for thigmotaxis, center entries, and center ambulation, with experiment as between factor.

For thigmotaxis, there was no main effect of experiment, $F(2, 138) = 2.00$, $p = .139$, $\eta_p^2 = .03$ [0.00; 0.08].

For center entries, there was a significant main effect of experiment, $F(2, 138) = 6.55$, $p = .002$, $\eta_p^2 = .09$ [0.02; 0.16]. Following up on the significant main effect with *post hoc* contrasts (using Tukey's method for correction for multiple comparisons) revealed a significant difference between experiment 2 vs. experiment 3, $t(138) = -3.57$, $p = .001$, but not between experiment 1 vs. experiment 2, $t(138) = 1.71$, $p = .205$, and between experiment 1 vs. experiment 3, $t(138) = -1.54$, $p = .274$. Participants in experiment 2 showed less center entries than participants in experiment 3.

For center ambulation, there was a significant main effect of experiment, $F(2, 138) = 7.44$, $p < .001$, $\eta_p^2 = .10$ [0.03; 0.17]. Following up on the significant main effect with *post hoc* contrasts (using Tukey's method for correction for multiple comparisons) revealed a significant difference between experiment 2 vs. experiment 3, $t(138) = -3.82$, $p = .001$, but not between experiment 1 vs. experiment 2, $t(138) = 1.89$, $p = .146$, and between experiment 1 vs. experiment 3, $t(138) = -1.58$, $p = .259$. Participants in experiment 2 showed less center ambulation than participants in experiment 3.

Taken together, participants in experiment 2 showed less center entries and center ambulation than participants in experiment 3.

Summary

Design of the open field did not affect anxiety and arousal ratings. In the experiment with the least objects spread across the open field (experiment 2), participants spent significantly less time in the center region and were, on average, closer to the wall, than participants in experiments 1 and 3, which both had more objects spread across the open field. Line crossing parameters indicated less center entries and center ambulation in experiment 2 compared to experiment 3, which is also likely a result of the number of objects spread across the open field.

In sum, open field design affected several behavioral parameters. The most likely reason for these differences between experiments is the number of objects spread across the open field. More objects (e.g., stones, flowers) in the open field center seem to be an incentive to enter this region.
